# Exploring the symptoms and sleep disorders associated with migraines in women of Syria: A cross‐sectional observational study

**DOI:** 10.1002/hsr2.2070

**Published:** 2024-04-23

**Authors:** Nafiza Martini, Tamam Hawa, Hussein Hamdar, Ali Alakbar Nahle, Majd Hanna, Douaa Albelal, Imad Addin Almasri, Ghassan Hamzeh, Stemosis Group of Data Collectors, Rinad Khudir, Qamar Alratta, Nour Abd Alaal

**Affiliations:** ^1^ Faculty of Medicine Damascus University Damascus Syrian Arab Republic; ^2^ Stemosis for Scientific Research Damascus Syrian Arab Republic; ^3^ Faculty of Medicine Hama University Hama Syrian Arab Republic; ^4^ Statistics Department Faculty of Economics Damascus University Damascus Syrian Arab Republic; ^5^ Faculty of Dentistry International University for Science and Technology (IUST) Damascus Syrian Arab Republic; ^6^ Faculty of Pharmacy Damascus University Damascus Syrian Arab Republic; ^7^ Faculty of Medicine Syrian Private University (SPU) Damascus Syrian Arab Republic

**Keywords:** cross sectional, female migraineurs, migraine symptoms, sleep disorders, Syria

## Abstract

**Background and Aim:**

Migraine is a prevalent neurological disorder characterized by recurring episodes of debilitating headache accompanied by associated symptoms and sleep disorders. This study aims to investigate migraine‐associated symptoms in female migraineurs within the Syrian population and the relation between migraines and sleep issues.

**Methods:**

A questionnaire‐based cross‐sectional observational study was conducted among the Syrian population. A total of 1009 women were enrolled in this study, including women without a history of migraine (Control group) and migraineurs (Case group) who had received a diagnosis of migraine from a hospital or private clinic. Data about migraine‐related symptoms, including tingling, visual disturbances, Nausea/Vomiting, and epileptic seizures as well as sleep‐related symptoms such as interrupted sleep, frequent awakenings, insomnia, snoring, and narcolepsy were gathered. Chi‐square test was used to examine the relation between migraines and sleep issues.

**Results:**

A total of 1009 women were enrolled in this study including 531 migraineurs and 478 healthy women. The study revealed that the most commonly experienced symptoms during migraine attacks were nausea/vomiting and visual disturbances, followed by tingling. Total Unduplicated Reach and Frequency analysis showed that visual disturbances and nausea/vomiting were the two most frequent symptoms that co‐occurred during migraine attacks. The study also demonstrated a significant relationship between snoring, insomnia, and narcolepsy with migraine (*p* = 0.038), with these sleep disorders being more prevalent among migraineurs.

**Conclusion:**

The findings indicate a significant association between migraines and sleep disorders, with migraineurs being at a significantly higher risk of experiencing poor sleep quality compared to healthy women. Addressing sleep disorders is crucial in managing patients with migraines. This study is the first of its kind in the Syrian population, providing valuable insights into the symptoms and sleep disorders associated with migraines in this population.

## 1 INTRODUCTION

Migraine is a prevalent and chronic neurological disorder characterized by recurring episodes of debilitating headache accompanied by associated symptoms, including aura, as well as reversible neurological and systemic symptoms.^1^ The pathophysiology of migraine is multifaceted and influenced by various factors, including rare genetic abnormalities. Migraine is one of the most common neurological disorders, affecting more than one billion people annually around the world me.^2^ According to the Global Burden of Diseases study in 2019, migraine cases have increased 16% from 1990. This concern is especially pronounced in low‐ to middle‐income countries such as Syria and exhibits a notable increase in prevalence among adolescents and young adults.^3^ Furthermore, migraines are common in women being affected three times more than men, especially during their childbearing years.^4^ Hormonal changes may play a role in the cause of migraines, as many women note that their migraine attacks occur in sync with their menstrual cycle.^1^, ^4^

The most characteristic symptoms associated with migraine include photophobia, phonophobia, cutaneous allodynia, and gastrointestinal symptoms such as nausea and vomiting. Additionally, patients can experience a variety of other neurological symptoms, such as vertigo, dizziness, tinnitus, and cognitive impairment.^2^, ^5^ Symptoms typically last from 4 to 72 h and can be debilitating. The pain is often, but not always, unilateral, sharp, worsens with exertion, and is accompanied by autonomic symptoms. Some patients also experience transient focal neurological deficits, often immediately before a headache attack, which is known as an aura.^2^

In addition, Migraine and epilepsy are distinct neurological diseases, but they share complex relationships. The clinical diagnosis of these disorders can be challenging due to overlapping symptoms. Seizures can trigger headaches, and there can be misdiagnosis between migraine aura and epilepsy, especially in cases of visual aura. Migraine with aura and epilepsy can also co‐occur, particularly in familial hemiplegic migraine.^6^

In terms of migraine comorbidities, Research indicated a significant bidirectional relationship between migraine and sleep disorders. Studies have demonstrated that insomnia can act as a risk factor for the onset of migraines, leading to increased pain intensity and impact.^7^, ^8^ Additionally, sleep issues like deprivation, excessive sleep, or irregular sleep patterns can contribute to the transition from episodic migraines to chronic migraines. The complex relationship between migraines and sleep disorders involves various neurophysiological processes. For example, chronic insufficient sleep has been associated with reduced tolerance to painful stimuli and changes that promote the chronicity of headaches. Hypothalamic and brainstem dysfunctions are commonly proposed as underlying mechanisms for the bidirectional relationship between migraines and insomnia.^9^, ^10^

Therefore, comprehending this connection can assist healthcare providers in developing more effective treatment strategies, ultimately leading to improved outcomes and a better quality of life. Given the absence of previous studies conducted within the Syrian population, to the best of our knowledge, the purpose of this paper was to examine the symptoms associated with migraines and examine the correlation between migraines and sleep disorders among women in Syria.

## 2 MATERIALS AND METHODS

### 2.1 Study design and population

This questionnaire‐based cross‐sectional observational study was conducted among the Syrian population for a period of 7 months from December 2021 to June 2022. The purpose of this study was to investigate the clinical characteristics of migraine headaches and associated symptoms among Syrian women and to examine the relation between migraines and sleep issues. A total of 1009 women were enrolled in this study. The study enrolled exclusively women without a history of migraine (Control group) as well as migraineurs (Case group) who had received a diagnosis of migraine from a hospital or private clinic and had experienced migraine symptoms such as severe headache pain, nausea, vomiting, and sensitivity to light and sound. Migraineurs who were excluded from the study had a history of head trauma or injury, long‐term use of medications that could impact migraine symptoms such as headaches NSAIDs and Acetaminophen,^11^ cognitive impairments that prevented them from completing the study interview, or other medical conditions that made it difficult to differentiate migraine disease from other diseases such as hypertension.^12^

At first, a self‐designed questionnaire was developed based on medical literature. To ensure the questionnaire's validity, a preliminary test was conducted with a small group of 10 women who had previously been diagnosed with migraines. This test aimed to assess the comprehensiveness and comprehensibility of the questionnaire. The feedback obtained from the participants helped in making adjustments to certain questions and adding new ones. The participants who took part in this pretest aided in the distribution of the questionnaires but were not included in the study. Following this, an invitation to partake in the research was published on social media platforms, primarily on Facebook due to its widespread usage. This was done to reach a larger audience and gather a diverse range of participants. Women who expressed interest in participating were contacted via phone to verify that they met the inclusion criteria. Once their eligibility was confirmed, they were given an overview of the study objectives, after which they received the questionnaire in the form of a Google form via messenger and WhatsApp. We programmed the survey link for single use by each respondent to prevent repeat survey participation.

### 2.2 Data collection and questionnaire

To gather detailed information, a questionnaire comprising three sections was utilized. The initial section aimed to gather sociodemographic data from both healthy women and those experiencing migraines. This included information such as age, marital status, education level, height/weight, and smoking habits. The second section focused on current migraine‐related symptoms, encompassing inquiries about nausea and vomiting, tingling sensations, and visual disturbances like flashes of light and blind spots. Additionally, we sought information regarding any history of epileptic seizures during migraine attacks. Also, we inquired about the occurrence of migraine during and/or outside menstruation. Lastly, the third section was applicable to both the Case and Control groups and addressed sleep‐related aspects. It encompassed questions about the sleep patterns (continuous or interrupted), the sleep timing (day or night), the presence of sleep disorders including insomnia, snoring, and narcolepsy, and awakenings type (Early and frequent awakenings).

### 2.3 Study measures and definition of terms

In terms of sociodemographic characteristics, the age of the participants was determined based on their age at the time of the study. Marital status was assessed to determine if participants were single, married, divorced, or widowed. Education level was categorized into different groups, including not educated, primary, secondary, university, and higher education. Smoking habits were classified as individuals who reported smoking cigarettes or using a hookah. Among cigarette smokers, further classification was done based on the number of cigarette packets smoked per day. The participants' weight was self‐reported in kilograms, while their height was self‐reported in meters.

Regarding symptoms, participants were asked about them using simple and easily understandable language to ensure comprehension and minimize bias. For tingling, participants were asked if they had ever experienced sensations like pins and needles, pricking, or numbness in their hands, feet, arms, or other parts of their bodies. Nausea and vomiting were assessed by inquiring about feelings of sickness or discomfort in the stomach, as well as the urge to vomit or the forceful ejection of stomach contents through the mouth. Visual disturbances were explored in three general categories: positive symptoms (e.g., zig‐zag lines, sparkles, dots, stars, and squiggles), negative symptoms (e.g., blind spots, tunnel vision, complete, or partial loss of vision), and altered/distorted visual symptoms (e.g., looking through water, heat waves, blurred vision, fractured vision, changes in color perception, size distortion, and misjudging distances). Regarding seizures, participants were asked if they had ever experienced sudden, uncontrolled body movements, changes in behavior, loss of awareness, changes in emotion, loss of muscle control, or shaking.

In terms of sleep‐related variables. As for sleep patterns (interrupted and continuous), interrupted sleep was considered if participants reported frequent awakenings during the night. Insomnia was identified if participants reported difficulty falling asleep or staying asleep. Narcolepsy was assessed by inquiring whether participants found it challenging to stay awake for extended periods and if they experienced sudden episodes of falling asleep.

### 2.4 Sample size

The sample size (*n*) was determined by Cochran's sample size formula with the assumption of 95.5% confidence level (*Z* = 2), *e* is the margin of error which is 10%, *p* is the (estimated) proportion of the population which has the attribute in question, and it equals 50% (or 0.5), and *q* is 1−*p*

$$n=\frac{z^{2}\mathrm{pq}}{{\mathrm{e}}^{2}}=\frac{{\left(2\right)}^{2}*0.5*0.5}{{\left(0.1\right)}^{2}}=100$$

The required sample size (*n*) for this study, applying the previous formula, is at least 100.

If we adopt a 5% margin of error. The sample size becomes 400.

$$n=\frac{z^{2}\mathrm{pq}}{{\mathrm{e}}^{2}}=\frac{{\left(2\right)}^{2}*0.5*0.5}{{\left(0.05\right)}^{2}}=400$$

Therefore, with a confidence of 95.5 and an error ranging from 5% to 10%, a sample size ranging from 100 to 400 individuals should be required. We had sought to have relied on the extent of the error of 5%, so depending on the previous equation, we must collect 400 questionnaires.^13^

### 2.5 Data analysis

Various statistical methods were employed to analyze the obtained results. Descriptive statistics including mean, standard deviation, frequencies, and percentages, were used to summarize variables such as age, marital status, and level of education. Additionally, Total Unduplicated Reach and Frequency (TURF) Analysis was conducted to identify the most common combinations of two and three migraine symptoms studied together. In terms of inferential statistics, Chi‐square tests were utilized to examine the differences in sleep‐related variables, including sleep disorders such as snoring and insomnia, between individuals with migraines and those without migraines. All statistical analyses were carried out using the Statistical Package for Social Sciences (SPSS) software, specifically version 25.

### 2.6 Ethics

The survey link was provided with an informed consent and an overview of the study, including its voluntary nature. Data collection was anonymous, with no identifying information being reported. Approval was obtained from the Ethics Committee of the Faculty of Medicine, Damascus University, Syria (14182).

## 3 RESULTS

### 3.1 Sociodemographic characteristic in control and case groups

A total of 1009 female, 531 migraineurs and 478 healthy women, participated in the study. Most of the migraineurs (61%) were single 3(12/511) and (82%) of healthy women were also single (381/465). Most of the sample held a university degree accounting for (397/523) in case group and (407/473) in control group. Additionally, it turned out that 676 both groups out of 989 were not smokers (68%). On average, Migraine patients had a weight of 63 kg, a height of 162 cm, and an age of 28 years versus a weigh of 59 kg, a height of 163 cm, and an age of 24 years for healthy group.

### 3.2 Migraine‐related symptoms in case group (migraineurs) (Tables 1 and 2)

**Table 1:** Frequencies and percentages of migraine related symptoms.

Variables	Category	Frequency	%
*Migraine‐related symptoms*			
Tingling	Absent	432	83.72%
	Present	84	16.28%
	Total	516	100%
Visual disturbances	Absent	215	41.67%
	Present	301	58.33%
	Total	516	100%
Epileptic seizures	Absent	471	91.28%
	Present	45	8.72%
	Total	516	100%
Nausea/vomiting	Absent	150	29.07%
	Present	366	70.93%
	Total	516	100%
*Migraine and menstrual cycles*			
Frequency of migraine attacks during menstrual cycle	Stable	169	31.80%
	Increase	362	68.20%
	Total	531	100%
Migraine attacks occurrence during and/or outside menstruation	Both during and outside menstruation	496	93.41%
	Solely during menstruation	35	6.59%
	Total	531	100%

**Table 2:** TURF analysis of Migraine‐related symptoms.

Variables	Reach and frequency	
	Reach	Pct of cases
Nausea/vomiting	366	%36.3
Visual disturbances	301	%29.8
Tingling	84	%8.3
Visual disturbances‐nausea/vomiting	497	%49.3
Nausea/vomiting‐tingling	401	%39.7
Tingling‐visual disturbances	325	%32.2
Visual disturbances‐nausea/vomiting‐tingling	506	%50.1

The study results indicated that out of 516 women, 432 (83.72%) did not experience tingling sensations during migraine attacks, whereas 84 women (16.28%) did. In addition, out of 516 women, 301 (58.33%) reported experiencing visual problems, while the remaining participants (41.67%) did not report any visual issues. Based on the study results, only a small number of women (45/516) experienced an epileptic seizure during their migraine attacks, while the vast majority of women (471/516) accounting for 91.28% did not. However, a significant majority of 366 out of 516 women (70.39%) experienced nausea and/or vomiting during their migraine attacks.

Based on TURF Analysis, nausea/vomiting was the most frequently reported symptom during migraine attacks, experienced by 36.3% of participants, followed by visual disturbances, which was the second most commonly reported symptom, experienced by 29.8% of participants.

According to the TURF analysis, the most frequently encountered symptoms that co‐occurred during migraine attacks were visual disturbances and nausea/vomiting. These two symptoms were reported together by 49.3% of women who experienced migraines. Moreover, visual disturbances, nausea/vomiting, and tingling were cited as the predominant cluster of symptoms commonly experienced concurrently during migraine attacks, with 50.1% of patients reporting the presence of these symptoms together.

In terms of menstrual cycles, it turned out that only 6.59% of all migraineurs suffered from migraine attacks solely during menstruation, whereas the majority (93.41%) experienced these attacks in both during and outside of menstruation.

### 3.3 Difference in sleep characteristic between migraineurs and healthy women (Table 3)

**Table 3:** Difference in sleep quality between migraineurs and healthy women.

Variables		478 Healthy women (control)		531 Migraineurs (case)		*p* Value^a^
		*n*	%	*N*	%	
Smoking status	Non smoker	338	72%	338	65%	0.006*
	One packet a day	22	5%	49	9%	
	Two packets a day	0	0%	3	1%	
	Hookah	110	23%	129	25%	
	Total	470	100%	519	100%	
Level of education	Uneducated	1	0.1%	0	0.0%	**–**
	Primary	1	0.1%	3	0.3%	
	Secondary	22	2.2%	32	3.2%	
	University	407	40.9%	397	39.9%	
	Higher education	42	4.2%	91	9.1%	
	Total	473	100%	523	100%	
Sleep patterns	Continuous	329	70%	258	49%	0.000*
	Interrupted	142	30%	265	51%	
	Total	471	100%	523	100%	
Sleep timing	Night	430	92%	493	94%	0.086
	Day	39	8%	29	6%	
	Total	569	100%	522	100%	
Awakening type	Early awakenings	286	64%	276	55%	0.004*
	Frequent awakenings	160	36%	227	45%	
	Total	446	100%	593	100%	
Sleep disorders	Insomnia	150	61%	263	71%	0.038*
	Snoring	20	8%	24	7%	
	Narcolepsy	75	31%	83	22%	
	Total	245	100%	370	100%	

^a^ Chi‐Square Tests;

* The Chi‐square statistic is significant at the 0.05 level.

Our findings indicate a significant statistical correlation between migraine and sleep patterns (interrupted or continuous sleep) (*p* < 0.001). The proportion of individuals with migraines experiencing interrupted sleep is notably higher compared to healthy women, with 265 out of 523 migraineurs (63%) reporting interrupted sleep, while only 142 out of 471 healthy women (30%) did. Conversely, healthy women tend to have more continuous sleep compared to migraine patients, with 329 out of 471 (70%) reporting continuous sleep, compared to 258 out of 523 (49%) among migraineurs. However, we did not find a significant correlation between sleep timing (day or night) and migraine in our results. Both the control and case groups reported similar findings (*p* = 0.09). Furthermore, frequent awakenings were significantly more common among migraineurs (*p* = 0.004), with 45% of patients (227/503) reporting it compared to only 35% of healthy women (160/446).

In terms of sleep disorders, our study demonstrated a significant relationship between snoring, insomnia, and narcolepsy with migraine (*p* = 0.04). These sleep disorders were more prevalent among migraineurs. Out of 413 women experiencing insomnia, 263 of them (64%) were migraineurs, while only 150 healthy women (36%) with insomnia were identified. Additionally, out of 44 women with snoring, 24 of them were migraine patients (55%) compared to 20 healthy women (45%) suffering from snoring. Regarding narcolepsy, out of 185 women reporting difficulty staying awake for extended periods, 83 were migraineurs, while 75 were healthy individuals.

## 4 DISCUSSION

The aim of this study was to investigate migraine‐associated symptoms in female migraineurs within the Syrian population and the relation between migraines and sleep issues. To the best of our knowledge, this study present the first of its kind in Syrian population. The study encompassed 1009 females, with 531 being migraineurs and 478 healthy women. Nausea/vomiting was the most frequently reported symptom during migraine attacks, followed by visual disturbances. Sleep patterns showed a correlation with migraines, with interrupted sleep more prevalent in migraineurs, while healthy women tended to have more continuous sleep. Sleep disorders like snoring, insomnia, and narcolepsy were more common among migraineurs.

Among the evaluated symptoms in our study, the findings revealed that the most commonly experienced symptoms during migraine attacks were nausea/vomiting and visual disturbances, followed by tingling. Based on TURF analysis, visual disturbances and nausea/vomiting were the two most frequent symptoms that co‐occurred during migraine attacks. Notably, 70.39% of the participants reported experiencing nausea/vomiting during their migraine episodes. However, previous studies have reported higher rates of nausea occurrence in migraine patients, with rates of 91.8% and 73%. The prevalence of vomiting was found to be lower in previous studies, with rates of 68.2% and 29%.^14^, ^15^ Although this shows an overlapping results between these studies, nausea and vomiting were major symptoms in migraine patients. Nausea is a characteristic symptom used to diagnose migraine, including the latest International Classification of Headache Disorders (ICHD) criteria.^16^ Reporting of nausea is also an essential component of accurate and reliable screening tools for migraine diagnosis,^17^ as it is a sensitive and specific indicator of the condition.

In our study, 58.33% of the participants reported visual disturbances, while previous studies reported rates of 73.2% and 44% for visual disturbances and blurred vision, respectively. Another study reported that photophobia was prevalent in 80% of patients.^14^, ^15^

Our study showed that the prevalence of epileptic seizures during migraine attacks was low, with only 8.72% of the study participants reporting experiencing a seizure during their migraine attacks. This is consistent with findings in other studies, where the occurrence of epilepsy in individuals with migraine has been reported to range from 1 to 17%, with a median of 5.9%, which is higher than the population prevalence of approximately 0.5−1%.^18^ However, the co‐occurrence of migraine among individuals with epilepsy is estimated to be between 8% and 24%.^19^ Furthermore, the likelihood of developing migraine is approximately twice as high in this population compared to the general population.^20^, ^21^ Although the occurrence is infrequent, this observation supports a link between migraine and epilepsy. The strong similarity between migraine and epilepsy, which supports the idea of a shared underlying mechanism, is further highlighted by the fact that certain antiepileptic drugs (AEDs) have been found to be beneficial in treating both conditions. Randomized controlled clinical trials have provided substantial evidence that divalproex sodium (valproate) and Topiramate are effective in preventing migraine attacks, and both drugs have been approved by the US Food and Drug Administration for this purpose.^22^ The gabapentinoids gabapentin and pregabalin may also be useful in migraine therapy,^23^, ^24^ in addition to other AEDs such as levetiracetam and zonisamide, which have been reported to have prophylactic effects against migraine.^25^, ^26^, ^27^

Our findings indicate a significant association between migraines and sleep disorders, with migraineurs being at a significantly higher risk of experiencing poor sleep quality compared to healthy women. All female patients reported a higher incidence of insomnia, snoring, narcolepsy, interrupted sleep, and frequent awakenings compared to the healthy group. These results are consistent with previous studies.^28^, ^29^, ^30^, ^31^, ^32^, ^33^, ^34^, ^35^ A study conducted in an Indian clinic found that 66.7% of migraine sufferers without aura had poor sleep quality, a percentage higher than nonmigraine participants.^36^ Additionally, research by Engstrom et al. revealed that migraine patients who had trouble sleeping reported increased daytime fatigue and lower pain tolerance.^37^

Sleep problems are often cited as triggers for migraine headaches. The relationship between sleep disorders and migraines is bidirectional, with disturbances in sleep patterns triggering migraines and migraines in turn impacting sleep negatively. For instance, insomnia not only increases the risk of migraine onset but also intensifies pain levels and the likelihood of chronic migraines.^9^ Habitual snoring is also recognized as a risk factor for chronic migraines.^38^

Although the association between migraines and sleep disorders is well‐documented, the underlying mechanisms and interactions are complex and not fully elucidated. Recent studies have identified specific brain structures and neurotransmitters involved in both migraines and normal sleep regulation, suggesting a potential causative role in the development of these disorders through shared nervous system pathways.^9^

Both the quantity and quality of sleep play crucial roles in overall health and well‐being. Migraine sufferers with poor sleep quality often exhibit symptoms of depression and anxiety. Furthermore, inadequate sleep is linked to various chronic health conditions such as heart disease, kidney disease, high blood pressure, diabetes, stroke, and obesity.^34^ Therefore, it is imperative to consider addressing sleep disorders when managing patients with migraines.

## 5 STRENGTH AND LIMITATION OF THE STUDY

There are some limitation of this study. People with more severe/disabling symptoms might have been more willing to engage in this study and, therefore, might be overrepresented. Recall bias is another potential limitation on this study due to the retrospective recollection retrieved by study participants and the questionnaire‐based nature of the study.

However, the study possesses several strengths, including a large sample size of 1009 women, consisting of both migraineurs and healthy individuals. This substantial sample size enhances the statistical power of the study, allowing for more reliable and accurate conclusions to be drawn. Additionally, the inclusion of both migraineurs and healthy women provides a comprehensive comparison group. Furthermore, a notable strength of this research is that it is the first of its kind conducted in Syria. By being the pioneering study in the country, it fills a significant gap in the existing literature and provides valuable insights into symptoms and Sleep Disorders Associated with Migraines.

## 6 CONCLUSION

The present study, carried out among a group of Syrian women, draws attention to the substantial influence of migraines, a widespread neurological condition that affects a significant number of individuals globally. Alongside shedding light on the typical symptoms linked to migraines, such as nausea/vomiting and visual disturbances, the investigation highlights the intricate connection between migraines and sleep disorders. The results reveal a notable correlation between migraines and compromised sleep quality, indicating that individuals with migraines are more likely to experience insomnia, snoring, narcolepsy, interrupted sleep, and frequent awakenings when compared to their healthy counterparts.

Recognizing and understanding this bidirectional connection between migraines and sleep disorders is crucial for healthcare providers in developing more effective treatment strategies. By addressing sleep issues as part of migraine management, there is potential for improved outcomes and a better quality of life for patients. The study's unique focus on the Syrian population fills a gap in the existing research, providing valuable insights into a region where such investigations were previously lacking.

## AUTHOR CONTRIBUTIONS

Nafiza Martini is a first author equally with Tamam Hawa, contributed to corresponding, drafting, editing, collecting data, and reviewing. The author reviewed and accepted the paper. Tamam Hawa contributed to collecting data, drafting, reviewing, and editing. The author reviewed and accepted the paper. Hussein Hamdar contributed to drafting, editing, and reviewing. The author reviewed and accepted the paper. Ali Alakbar Nahle contributed to drafting, editing, and reviewing. The author reviewed and accepted the paper. Majd Hanna contributed to drafting, editing, reviewing, and bibliography. The author reviewed and accepted the paper. Douaa Albelal contributed to drafting, editing, and reviewing. The author reviewed and accepted the paper. Imad Addin Almasri contributed to Data analysis, collecting data, reviewing, and editing. The author reviewed and accepted the paper. Ghassan Hamzeh is the supervisor, contributed to editing and reviewing. The author reviewed and accepted the paper. Stemosis Group of Data collectors: Collected data. All authors reviewed and accepted the paper.

## CONFLICT OF INTEREST STATEMENT

The authors declare no conflict of interest.

## ETHICS STATEMENT

The Ethical Committee approved this study in the Faculty of Medicine at Damascus University, Syria with the number (14182). The study complied with the principles of the Helsinki Declaration.

## TRANSPARENCY STATEMENT

The lead author Nafiza Martini affirms that this manuscript is an honest, accurate, and transparent account of the study being reported; that no important aspects of the study have been omitted; and that any discrepancies from the study as planned (and, if relevant, registered) have been explained.

## Data Availability

The data sets collected and analyzed during the current study are not publicly available. Some restrictions apply to the availability of these data but are available from the corresponding author on reasonable request.
